# IBD Camp Oasis: A look at Participants’ Social-Emotional Well-Being and Protective Factors During Camp and Beyond

**DOI:** 10.1093/crocol/otad042

**Published:** 2023-08-24

**Authors:** Namita Singh, Steven J Steiner, Rebecca Fauth, Danyel Moosmann, Janis Arnold, Abdul Elkadri, Daniel Marinoni, Laurel Molloy, Becky Johnson Rescola, Jeanne Tung, Elizabeth C Utterson

**Affiliations:** Co­Director of Pediatric IBD Center, Assistant Professor of Pediatrics, University of Washington–School of Medicine, Seattle, USA; Professor of Pediatrics, Riley Hospital for Children, Indiana University School of Medicine, Indianapolis, USA; Research Associate Professor, Co-Director, Tufts Interdisciplinary Evaluation Research (TIER), Eliot­Pearson Department of Child Study and Human Development, Tufts University, Medford, USA; Project Manager | Tufts Interdisciplinary Evaluation Research (TIER), Tufts University, Medford, USA; Clinical Social Worker, Division of Gastroenterology, Hepatology, Boston, USA; Pediatric Gastroenterology, Assistant Professor, the Medical College of Wisconsin, Milwaukee, USA; Associate Director, Camp Oasis, Crohn’s & Colitis Foundation, New York, USA; Technical Advisor, Camp Oasis, Crohn’s & Colitis Foundation, New York, USA; Vice President, Education & Community Engagement, Crohn’s & Colitis Foundation, New York, USA; Associate Professor, Pediatric Gastroenterology, the University of Oklahoma College of Medicine, Oklahoma City, USA; Associate Professor, Pediatrics, Division of Pediatric Gastroenterology Director of Procedures, Washington University Physicians, St Louis, USA

**Keywords:** camp, IBD, Crohn’s disease, ulcerative colitis, pediatric IBD, well-being, mental health

## Abstract

**Background:**

Camp Oasis is an annual week-long camp serving children with inflammatory bowel disease (IBD) and hosted by the Crohn’s and Colitis Foundation. Youth with IBD are at increased risk for mental health challenges, with Camp Oasis potentially mitigating these risks. The aim of this study is to measure change in and predictors of social-emotional well-being and protective factors of self-worth as a result of attending Camp Oasis.

**Methods:**

Between 2012 and 2019, a voluntary survey was administered to participants and their caregivers to reflect on their perceptions of social/emotional well-being and protective factors related to chronic disease. *T*-tests compared change in participants’ and caregivers’ perceptions before and after camp; path analyses examined the key predictors of social-emotional well-being.

**Results:**

A total of 6011 online surveys were analyzed. Participants and caregivers reported consistently positive perceptions of participants’ experiences during and after camp. Significant improvements in confidence, independence, activity, comfort around others, being more open about disease, and taking medication as expected were observed. Being new to Camp Oasis was one of the strongest predictors of both disease-related self-efficacy and social connections after camp.

**Conclusions:**

The uniformly high rates of participants’ perceptions during camp suggest camp is a life-changing experience for youth with IBD, reduces disease-related stigma, and enhances confidence and social skills. Participants’ positive experiences appear to foster notable benefits after camp in terms of openness, their sense of belonging, connections, and confidence.

## Graphical Abstract

**Graphical Abstract:**
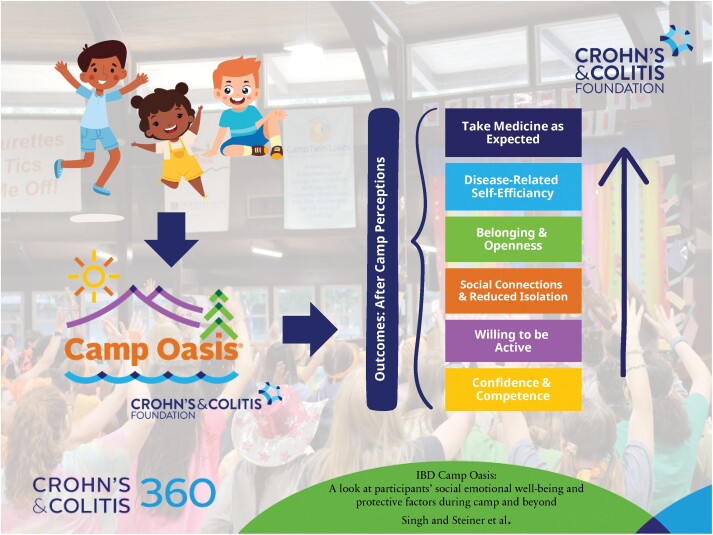

## Introduction

Approximately 70 000 to 80 000 youth in the United States are currently diagnosed with inflammatory bowel disease (IBD), consisting of Crohn’s disease and ulcerative colitis.^1^ These youth are at increased risk for depression, anxiety, and social-emotional difficulties, which may continue into adulthood.^2–4^ The etiology of these difficulties may be attributed to disease symptomatology and challenges with or side effects from treatment, but also stigma and social isolation experienced by youth with IBD.^5^

For more than 20 years, Camp Oasis, a week-long camp administered by the Crohn’s and Colitis Foundation across 12 locations in the United States, has provided an overnight summer camp experience for youth with IBD. Each camp provides traditional camp activities under the supervision of medical staff and adult volunteers, most with professional and/or personal experience with IBD. This residential camp setting provides a safe, fun experience for children entering second grade up to and including rising high school seniors. Campers are provided opportunities to participate in small cohorts (cabins), larger groups, and all camp activities each day, providing peer support as well as opportunities to interact with other kids of all ages. Camp Oasis participants must have a diagnosis of IBD and be medically stable at the time of camp. Each year, Camp Oasis creates an environment where the experiences of IBD are normalized for all participants.

By building disease-related resilience and self-efficacy, providing opportunities for socialization with peers who have IBD, and offering enrichment activities in a safe and inclusive environment, Camp Oasis may mitigate the likelihood of youth experiencing longer-term psychosocial difficulties. Camp Oasis fosters a sense of independence and allows children and teens to manage medications, food choices, and activities under the supervision of volunteer medical staff and adult leaders. Volunteers serve as both educators and mentors throughout the camp week to empower kids and teens in learning more about managing IBD.

Camp Oasis elicits camp participants’ perceptions of camp via an optional anonymous online survey administered annually to caregivers and camp participants. The aim of this study is to measure change in and examine predictors of social-emotional well-being and protective factors as a result of attending Camp Oasis.

## Materials and Methods, and Ethical Considerations

### The Annual Camp Oasis Survey

After participating in Camp Oasis, participants and caregivers are asked to complete an online survey. The survey focuses on participants’ perceptions and experiences of social-emotional well-being and protective factors related to chronic disease before, during, and after camp. The survey is distributed by e-mail only to parents/caregivers of camp attendees approximately 2 weeks after the conclusion of the camp session. No direct contact was made with the campers themselves. Participants (and their caregivers) who attend camp more than once are resurveyed each year that they attend. The survey was only available in English.

To date, survey data have mainly been used for an internal evaluation of recruitment efforts and to assess potential camp impacts and inform approaches on a smaller scale (eg, examining how data compares from one year to the next); the current report analyzes the data to understand the perceived impact of Camp Oasis on participants more broadly to evaluate larger trends. Given the goal of the survey data for program improvement, and the fact that survey respondents remained anonymous without identifying attributes, IRB approval was determined not to be necessary. No formal consent or assent was obtained during the collection of the survey data.

### Measures

#### Camp oasis outcomes

**Figure 1.**
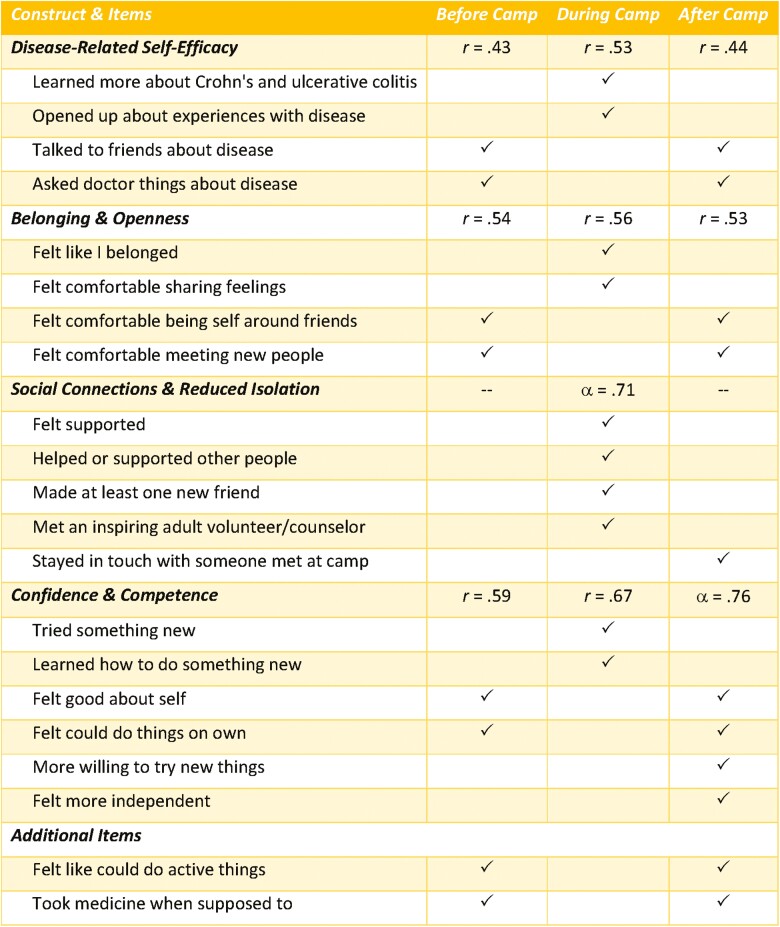
Description of camp oasis social-emotional well-being and protective factors constructs and Items. Checks indicate for which timepoint the participant or caregiver was asked to provide a rating. *r *= Pearson correlation coefficient for scales with 2 items, α = Cronbach’s alpha for scales with at least 3 items.

As seen in Figure 1, many of the items asked at each timepoint were comparable. Items were grouped into 4 overarching constructs: (1) *confidence and competence*, (2) *social connections and reduced isolation*, (3) *belonging and openness*, and (4) *disease-related self-efficacy*. Constructs were devised based on the evaluation by an advisory group comprised of physicians, nurses, and mental health specialists. Cronbach’s alphas were computed to assess the internal consistency of items within each construct; correlations were used if constructs comprised just 2 items. All alphas and correlations were acceptable, except for 2 items (ie, “Felt like could do active things like playing sports and running around,” “Took medicine when supposed to”) that were unrelated to other items; these items were not incorporated into a construct. For the remaining items, summary variables representing each of the 4 constructs were created by computing the mean of the items included in the respective construct (Figure 1).

#### Background variables

To understand and contextualize variation in participants’ social-emotional well-being and protective factors, surveys included a range of background variables including respondent type, self-identified gender, age, return/new participant, time since diagnosis, status of having met an adult/peer with IBD prior to attending camp, having slept away from home without family prior to attending camp, having attended another overnight camp prior.

Average ratings for each of the Camp Oasis social-emotional well-being and protective factors before, during, and after camp were evaluated. Each item was rated on a 4-point Likert scale, with a score of 4 indicating a more favorable perception.

### Analytic Strategy

The primary analysis focuses on participants’ and caregivers’ perceptions during and after camp using descriptive statistics (means, standard deviations). Dependent *t*-tests examined change in perceptions before versus after camp. Finally, structural equation model was used to explore the strongest predictors of participants’ social-emotional well-being and protective factors after camp, including participants’ background characteristics and participants’ and caregivers’ perceptions before and during camp as predictors.

## Results

**Figure 2.**
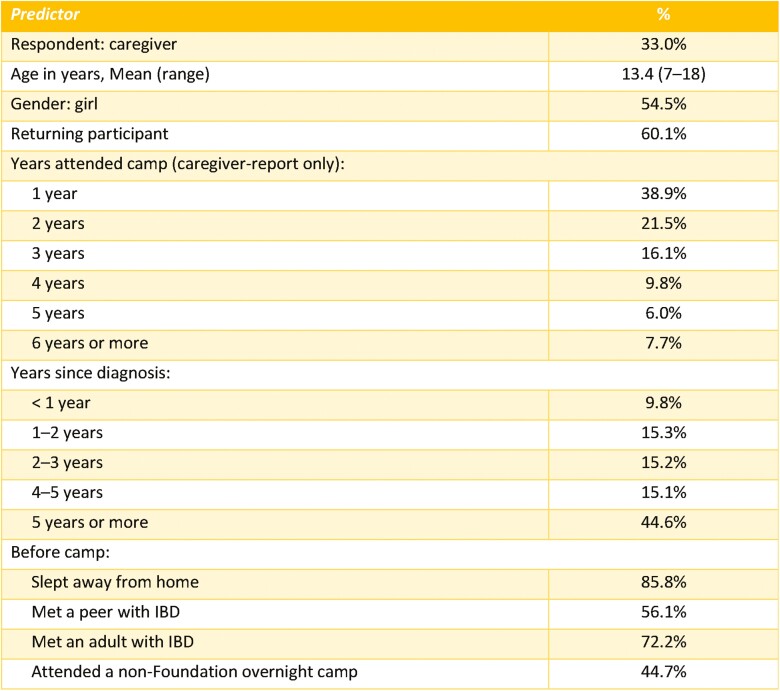
Background characteristics of camp oasis participants 2012–2019 (*n* = 6011).

A total of 6011 online surveys (*n *= 1986 camp participants, *n *= 4025 caregivers) were collected from 2012 to 2019. Characteristics of respondents and caregivers are described in Figure 2.

### Participants’ Social-Emotional Well-Being and Protective Factors

Participants’ perceptions of their social-emotional well-being and protective factors were favorable across all timepoints included in the survey. Notably, participants and caregivers reported consistently high perceptions of participants’ experiences during and after camp, with more than 90% of survey respondents agreeing with each item in the survey.

When considering participants’ experiences during camp, participants’ and caregivers’ ratings were uniformly high (range = 3.50–3.82), indicating camp is a place where participants can try to new things, feel supported and make friends, garner new friendships and social supports, and learn more about their disease.

**Figure 3.**
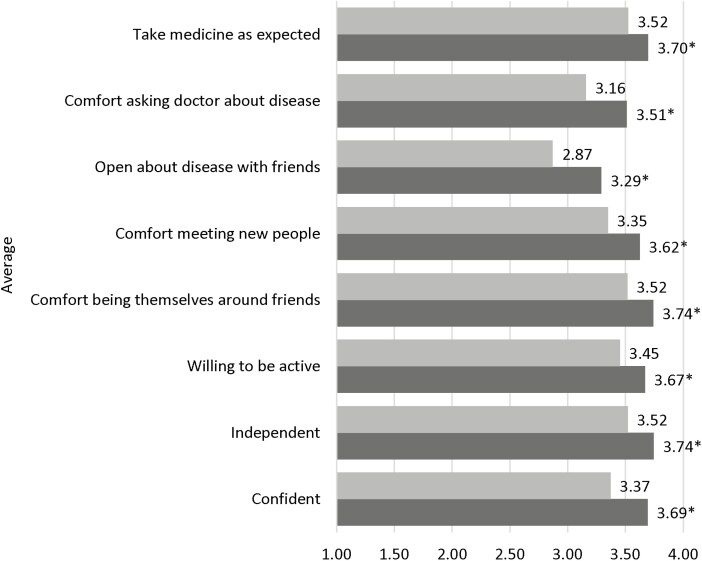
Camp oasis outcomes: Before camp (light bars) versus after camp (dark bars) *n* = 5827. Difference between before camp and after camp values was statistically significant (*P < .05) for all 8 social-emotional and protective factors.

Dependent *t*-tests compared the means of participants’ and caregivers’ perceptions of the outcomes before camp and after camp at the item-level (for each item that overlapped) to examine change over time in perceptions. Uniformly, the eight social-emotional well-being and protective factors were statistically greater (*P* < .05) after camp (dark bar) relative to before (light bar). (Figure 3).

### Path Analysis Results

**Figure 4.**
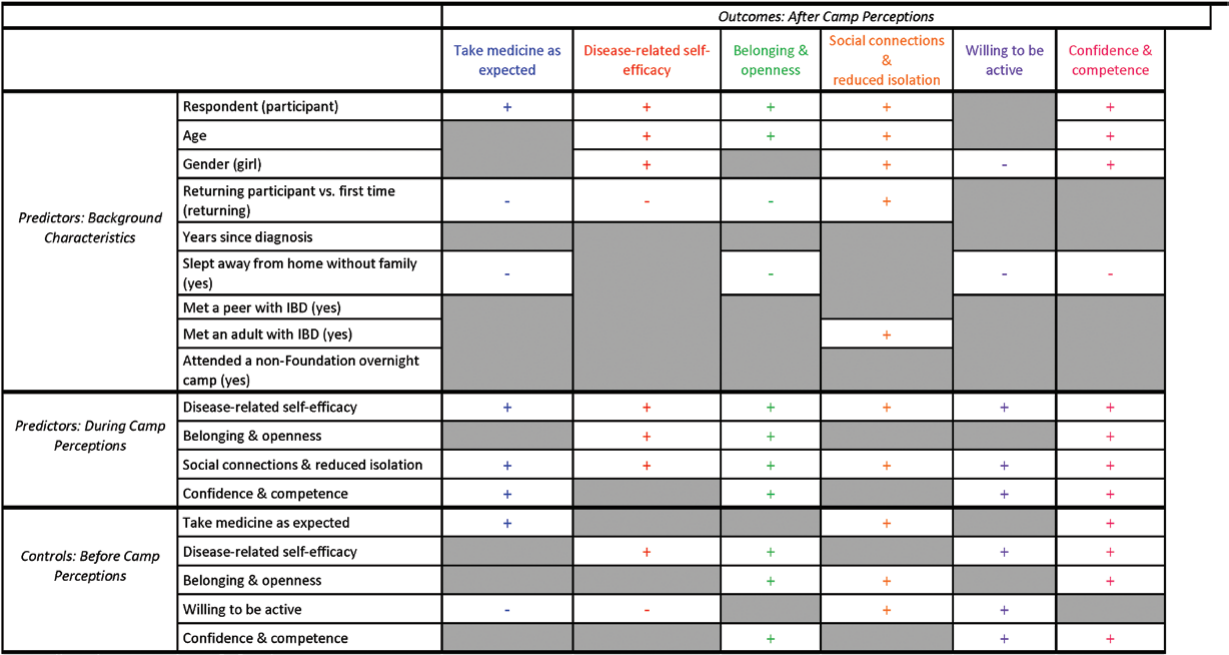
Summary of the path analysis results.** **+ denotes statistically significant (*P* < .05) positive association between predictor and outcome;—denotes statistically significant (*P* < .05) negative association between predictor and outcome. For categorical variables (eg, gender, returning camper vs. first time), the category in parentheses is the base category, so, for example, a positive association between gender and an outcome variable indicates that girls had higher scores on the outcomes; whereas a negative association indicates boys had higher scores. Gray cells indicate no association between the respective predictor and outcome.

Findings from the structural equation model are presented in Figure 4. Results are summarized by outcome construct.

#### Take medicine as expected

Compared to returning participants and participants who had previously slept away from home without family, new participants and participants who had not previously slept away from their families were more likely to take their medicine as expected after camp. Perceptions related to experiences during camp, including *disease-related self-efficacy* (eg, learning more about IBD and opening up about their experiences with IBD), *social connections and reduced isolation* (eg, feeling supported, helping/supporting others, making a friend, meeting an adult who inspired them), and *confidence and competence* (eg, trying/learning something new), were positively associated with *taking medicine as expected* after camp. Participants who reported less willingness to be active before camp exhibited higher scores on this outcome.

#### Disease-related self-efficacy

Girls, older participants, and new participants were more likely to have higher perceptions of *disease-related self-efficacy* after camp (eg, talking to friends about their IBD and feeling comfortable asking their doctor about IBD). Perceptions related to experiences during camp, including *disease-related self-efficacy*, *belonging and openness* (eg, feeling like they belonged and comfortable sharing their feelings), and *social connections and reduced isolation* were positively associated with perceptions of *disease-related self-efficacy* after camp. Participants who were less *willing to be active* before camp had higher perceptions of *disease-related self-efficacy* after camp. Participants’ perceptions of disease-related self-efficacy during camp and being new to Camp Oasis (3.44 new vs. 3.38 returning) were the strongest predictors of disease-related self-efficacy after camp.

#### Belonging and openness

Older participants, new participants, and participants who had not previously slept away from their families were more likely to have higher perceptions of *belonging and openness* after camp (eg, feeling comfortable being themselves around their friends and meeting new people). Perceptions related to experiences during camp, including *disease-related self-efficacy*, *belonging and openness*, *social connections and reduced isolation*, and *confidence and competence* were positively associated with perceptions of belonging and openness after camp. Youth’s experiences of social connections and reduced isolation during camp was the strongest predictor of their sense of belonging and openness after camp. Youth for whom Camp Oasis was their first experience sleeping away from home also reported favorable perceptions of belonging and openness. Camp Oasis’s activities that promote youth’s social connections helps them feel a greater sense of belonging and openness with others after camp.

#### Social connections and reduced isolation

Older participants, girls, returning participants, and participants who had met an adult with IBD before attending camp were more likely to have higher perceptions of *social connections and reduced isolation* after camp (eg, staying in touch with someone they met after camp). Perceptions related to experiences during camp, including *disease-related self-efficacy* and *social connections and reduced isolation* were positively associated with perceptions of *social connections and reduced isolation* after camp.

Participants’ gender (girl 3.51 vs. 3.06 boy), increase in age, being a returning participant (3.37 returning vs. 3.21 new), and campers’ social connections during camp were the strongest predictors for social connections after camp.

#### Willingness to be active

In comparison to girls and participants who had previously slept away from home without family, boys and participants who had not previously slept away from their families were more likely to be *physically active* after camp (eg, playing sports and running around). Perceptions related to experiences during camp, including *disease-related self-efficacy*, *social connections and reduced isolation*, and *confidence and competence* were positively associated with *willingness to be active* after camp.

#### Confidence and competence

Older participants, girls, and participants who had not previously slept away from their families were more likely to have higher perceptions of *confidence and competence* after camp (eg, feeling good about themselves and more independent; willing to try to new things). Perceptions related to experiences during camp, including *disease-related self-efficacy*, *belonging and openness*, *social connections and reduced isolation*, and *confidence and competence* were positively associated with perceptions of *confidence and competence* after camp.

Participants’ favorable experiences during camp, especially those that enhanced their disease-related self-efficacy, confidence and competence, and social connections and reduced isolation, were the strongest predictors of their confidence and competence after camp.

## Discussion

Based on survey findings, participants and caregivers reported consistently positive perceptions of participants’ experiences during and after camp. Notably, participants’ sense of *belonging* and openness and *connections and reduced isolation* during camp and participants’ *compliance taking medicine*, *willingness to be active*, and *belonging and openness* after camp were most favorable. Our study was limited by having a survey which was only available in English, and only available by e-mail, which may have limited participation from a small number of important participants. However, Camp Oasis clearly promoted a strengthened sense of belonging for its participants, many of whom may have experienced social challenges and feelings of isolation in their lives.

Participants’ experiences during camp were consistently and favorably associated with their experiences after camp. Of note, participants’ *social connections* during camp were among the strongest predictors of youth’s *sense of belonging*, *social connections*, and *confidence* after camp. Participants’ perceptions of *disease-related self-efficacy* during camp was a strong predictor of both *disease-related self-efficacy* and youth’s *confidence and competence* after camp. These findings suggest that Camp Oasis leads to a positive shift in participants’ social worlds, ways of relating to their peers, and ability to cope with their disease—findings with potential long-term benefits as they move into early adulthood.

With few exceptions, older participants, girls, new participants, and participants who had not slept away from home without family before attending camp reported more favorable psychosocial adjustment following Camp Oasis participation. Notably, being new to Camp Oasis was one of the strongest predictors of both *disease-related self-efficacy* and s*ocial* connections after camp. The whole of these findings suggest that Camp Oasis is most impactful and life-changing for youth who have not previously had a comparable experience, a true testament to the importance of Camp Oasis for youth experiencing IBD.

While new participants typically demonstrated more favorable outcomes, perceptions of participants’ *social connections and reduced isolation* after camp were higher among returning participants, suggesting that participants are making, maintaining, and sustaining friendships over the years. And, relative to girls, boys’ *willingness to be active* was higher, suggesting that Camp Oasis provides a safe space for participants, especially boys, to engage in physical activity.

Several other studies have evaluated the effect of summer camp on pediatric patients with IBD. Shepanski et al^6^ found improved quality of life scores, including bowel symptoms and social functioning domain scores, but no change in anxiety in 61 patients following one week of camp. Using open-ended questions, Salazar et al^7^ observed benefit from attending a camp for pediatric IBD, and broadly summarized the answers into themes of “Kids Like Me,” “Not the Only One,” and “Perspectives on IBD.” McCombie et al^8^ surveyed 36 patients who attended summer camp for children in New Zealand, and determined that camp improved confidence, acceptance, and overall quality of life, and that meeting their fellow campers was the most beneficial aspect of camp. The same group of investigators later assessed campers at baseline, 1 month after, and 6 months after attending summer camp. Although mean quality of life scores did not increase after 1 or 6 months (except for body image sub-score at 6 months), disease specific knowledge was enhanced by attending camp and maintained at 6 months following camp.^9^

## Conclusion

This analysis is the largest of its kind in describing effects of IBD camp on children with IBD. This was intended to help Camp Oasis identify their potential impact on youth with IBD and to inform future programming and evaluation efforts. The analysis provides preliminary evidence on the role Camp Oasis has in enhancing the lives of youth with IBD. The findings are of interest to the larger community of caregivers, medical practitioners, and patients with IBD and hopefully generate enthusiasm for the importance of camp for participants and families.

## Data Availability

Data not publicly available.
